# Supplementary material for: AICAR attenuates postoperative abdominal adhesion formation by inhibiting oxidative stress and promoting mesothelial cell repair
Source: PLoS One. 2022 Sep 1;17(9):e0272928. doi: 10.1371/journal.pone.0272928 (PMC9436141; doi:10.1371/journal.pone.0272928)
Supplement: Attachment — Submitted filename: Response to Reviewers.docx [file pone.0272928.s002.docx]

Dear Editor Hossain,

Thank you very much for determining that our manuscript titled **“*AICAR attenuates postoperative abdominal adhesion formation by inhibiting oxidative stress and promoting mesothelial cell repair*”** is potentially acceptable for publication in ***PLOS ONE***, pending a satisfactory revision of the paper in accordance with the enclosed reviewers' comments. We sincerely appreciated the reviewers’ comments and felt encouraged by their positive feedback regarding our manuscript. The remaining concerns of the reviewers and their suggestions for improving the manuscript have been carefully addressed in our revised manuscript. Below, we provide a point-by-point response to the comments. For clarity, we present those requests in italics, followed by our responses.

We believe that the revisions following the reviewer’ advice have substantially improved the manuscript, and we hope that the revised manuscript is suitable for publication in ***PLOS ONE.***

Looking forward to hearing from you soon.

With kind regards,

Yours sincerely,

Fei Xue, Ph.D., M.D.

The Second Department of General Surgery, Shaanxi Provincial People's Hospital, 256 West Youyi Road, Xi'an, Shaanxi, China, 710061

E-mail address: xfx1129@126.com

**Reviewer #1 (Reviewer Comments to the Author)**

**Response to** **Reviewer #1**

Many thanks for your expert comments and suggestions for improving our manuscript. We have followed your advice and have improved the presentation of the manuscript accordingly.

1. *Multiple studies indicated that p-eNOS expression and nitric oxide (NO) production were increased by in vivo administration of AICAR; please include results of NO and p-eNOS levels in your in vivo study groups. This data will further justify the effect of AICAR on oxidative stress.*

**Response:** Thank you very much for your advice, to further demonstrate the effect of AICAR on the expression NO and p-eNOS, we had both detected the NO level by ELISA and the p-eNOS by IHC, however, we had fail the for the IHC staining of p-eNOS. However, the NO level had been increased by the AICAR treatments. As shown in Figure 3C.

2. *As the authors indicated in the discussion section, “Peritoneal mesothelial cell mesenchymal transition (MMT) is an important process in fibrosis” please include the results of* *α-SMA and vimentin expression levels in your in vivo study groups (like you did for Nrf2).*

**Response:** Thank you very much for your advice, we have added the α-SMA IHC staining for each group, as shown in figure (4A3 and 4D). The result showed that AICAR treatment can protect the integrity of peritoneal mesenchymal cell and inhibits tissue fibrogenesis. Due to a lack of time, we did not preformed the vimentin IHC staining.

3. *In Figure 5D, the number of visible cells in the TGF-β group is way less than in other groups, which dismantled the validity of the quantitative data; please replace this image with the better one and recalculate the graph accordingly.*

**Response:** Thank you very much. We chose fewer cell image fields in order to have a better view of cell morphological changes and fluorescence changes. Since the peritoneal mesothelial cells had turned into fibers and became smaller, they appeared to have fewer cells, so we have replaced with another one.

**Reviewer #2 (Reviewer Comments to the Author)**

**Response to Reviewer #2**

Many thanks for your expert comments and suggestions for improving our manuscript. We have followed your advice and have improved the presentation of the manuscript accordingly.

1. *Indicate the full name of AICIR (5-aminoimidazole-4-carboxyamide ribonucleoside) in the Material and Methods section and the purchasing information (company, purity, etc.).*

**Response:** Thank you for for carefully reminding, we purchased the AICIR from cell signaling technology, USA, purity >99%.

2. *Please include brief information about Nair’s scoring system and add a reference.*

**Response:** Thank you very much, we have added the Nair’s scoring system in our manuscript and added the citation.

3. *Please includes the purchasing information for the MDA detection kit.*

**Response:** Thank you for your advice, we have added the related information in our manuscript.

4. *In Figure 5D, the authors measured the transcriptional (mRNA) levels of E-cadherin, a-SMA, and Vimentin. Please indicate it in the figure, e.g., Relative mRNA expression of E-cadherin.*

**Response:** Thank you for your reminding, we have revised them.

5. *Please italicize the words in vivo and in vitro in the manuscript. The statistically significant symbol “P” should always indicate by a small letter (and italicize it); please correct it in the manuscript.*

**Response:** Thank you very much for your reminding, we have revised them.

6. *In Figure 3A, please replace “Relative ROS expression (%)” with “Relative ROS levels (%)” and subsequently correct the figure legend.*

**Response:** Thank you very much for your reminding, we have revised them.

**Reviewer #3 (Reviewer Comments to the Author)**

**Response to Reviewer #3**

1. *In the abstract-back ground section should describe solely context of the research rather than describing results and concluding remark.*

**Response:** Thank you for your suggestion, we have revised it.

2. *The authors conclude that AMPK activator AICAR inhibits inflammation and oxidative stress in the adhesion tissue. Thus, the author should first confirm AMPK and inflammatory signaling (i.e TGF-β and* *NF-kb) activation in the adhesion tissue by* *western blotting. All they evaluated to confirm* *anti-oxidative effects of AICAR were ROS and MDA. Thus reviewer is interested to see* *Sod, catalase and Gpx expression in the adhesion tissue.*

**Response:** Thank you for your suggestion. We have shown the TGF-β1 concentration result after different treatments in Figure 2C, and we can also show you the time-course result we did to confirm that TGF-β1 accumulation in adhesion tissue. Please see in below. But for this part of result, we don’t want to added in the figures.

Since NF-kb signaling pathway activation has been widely confirmed in adhesion tissue[1-4]. So, we don’t think we need to repeat this part again.

1. Chen S, Jiang S, Zheng W, Tu B, Liu S, Ruan H, Fan C. RelA/p65 inhibition prevents tendon adhesion by modulating inflammation, cell proliferation, and apoptosis. Cell Death Dis. 2017 Mar 30;8(3):e2710. doi: 10.1038/cddis.2017.135. PMID: 28358376; PMCID: PMC5386538.
2. Yang L, Lian Z, Zhang B, Li Z, Zeng L, Li W, Bian Y. Effect of ligustrazine nanoparticles on Th1/Th2 balance by TLR4/MyD88/NF-κB pathway in rats with postoperative peritoneal adhesion. BMC Surg. 2021 Apr 26;21(1):211. doi: 10.1186/s12893-021-01201-7. PMID: 33902534; PMCID: PMC8077798.
3. Jiang ZL, Fletcher NM, Diamond MP, Abu-Soud HM, Saed GM. Hypoxia regulates iNOS expression in human normal peritoneal and adhesion fibroblasts through nuclear factor kappa B activation mechanism. Fertil Steril. 2009 Feb;91(2):616-21. doi: 10.1016/j.fertnstert.2007.11.059. Epub 2008 Feb 20. PMID: 18281043; PMCID: PMC2812021.
4. Li L, Frei B. Iron chelation inhibits NF-kappaB-mediated adhesion molecule expression by inhibiting p22(phox) protein expression and NADPH oxidase activity. Arterioscler Thromb Vasc Biol. 2006 Dec;26(12):2638-43. doi: 10.1161/01.ATV.0000245820.34238.da. Epub 2006 Sep 14. PMID: 16973969.

For Sod, catalase and Gpx expression, we have added Sod, catalase and Gpx activity analysis in Figure 3D.

3. *They have shown inhibitory effects of AICAR on fibrosis and adhesion formation. Therefore, it would be interesting to see the protein abundance of collagen1, α-SMA, integrins and E-cadherin in the adhesion tissue of the post-operative rats.*

**Response:** Thank you for your advice, we have added the α-SMA IHC staining of post-operative rats in our manuscript, as shown in figure (4A3 and 4D).
